# Suppression of TGFβR-Smad3 pathway alleviates the syrinx induced by syringomyelia

**DOI:** 10.1186/s13578-023-01048-w

**Published:** 2023-05-29

**Authors:** Sumei Liu, Longbing Ma, Boling Qi, Qian Li, Zhiguo Chen, Fengzeng Jian

**Affiliations:** 1 Department of Neurosurgery, China International Neuroscience Institute, Xuanwu Hospital Capital Medical University, 45 Changchun Street, Beijing, 100053 China; 2 Cell Therapy Center, Xuanwu Hospital Capital Medical University, 45 Changchun Street, Beijing, 100053 China; 3 Spine Center, China International Neuroscience Institute (CHINA-INI), Beijing, China; 4 Lab of Spinal Cord Injury and Functional Reconstruction, Xuanwu Hospital, Capital Medical University, Beijing, China; 5 Research Center of Spine and Spinal Cord, Beijing Institute of Brain Disorders, Capital Medical University, Beijing, China; 6 National Center for Neurological Disorders, Beijing, China

**Keywords:** Spinal cord, Central canal, Ependymal cells, Syrinx

## Abstract

**Background:**

Syringomyelia is a cerebrospinal fluid (CSF) disorder resulted in separation of pain and temperature, dilation of central canal and formation of syrinx in central canal. It is unclear about mechanisms of the dilation and syrinx formation. We aimed to investigate roles of ependymal cells lining central canal on the dilation, trying to reduce syrinx formation in central canal.

**Methods:**

We employed 78 Sprague–Dawley (SD) rats totally with syringomyelia to detect the contribution of ependymal cells to the dilation of central canal. Immunofluorescence was used to examine the activation of ependymal cells in 54 syringomyelia rat models. BrdU was used to indicate the proliferation of ependymal cells through intraperitoneal administration in 6 syringomyelia rat models. 18 rats with syringomyelia were injected with SIS3, an inhibitor of TGFβR-Smad3, and rats injected with DMSO  were used as control. Among the 18 rats, 12 rats were used for observation of syrinx following SIS3 or DMSO administration by using magnetic resonance imaging (MRI) on day 14 and day 30 under syringomyelia without decompression. All the data were expressed as mean ± standard deviation (mean ± SD). Differences between groups were compared using the two-tailed Student’s *t*-test or ANOVA. Differences were considered significant when *p < 0.05.

**Results:**

Our study showed the dilation and protrusions of central canal on day 5 and enlargement from day 14 after syringomyelia induction in rats with activation of ependymal cells lining central canal. Moreover, the ependymal cells contributed to protrusion formation possibly through migration along with central canal. Furthermore, suppression of TGFβR-Smad3 which was crucial for migration reversed the size of syrnix in central canal without treatment of decompression, suggesting TGFβR-Smad3 signal might be key for dilation of central canal and formation of syrinx.

**Conclusions:**

The size of syrinx was decreased after SIS3 administration without decompression. Our study depicted the mechanisms of syrinx formation and suggested TGFβR-Smad3 signal might be key for dilation of central canal and formation of syrinx.

**Supplementary Information:**

The online version contains supplementary material available at 10.1186/s13578-023-01048-w.

## Background

Syringomyelia is a disease characterized by cystic cavities composed of fluid similar to cerebrospinal fluid (CSF) or extracellular fluid in the spinal cord, representing dilation of the central canal, damaging the spinal nerves and leading to weakness, pain, and paralysis [1–3]. Various factors lead to syringomyelia, such as compression of spinal tissue, spinal tumor, arachnoiditis, and choroid plexus in the central canal [4, 5]. Syringomyelia is most often associated with Chiari malformation, which results from CSF outflow from the fourth ventricle, diverting the CSF pulse waves into the central canal [6]. This theory is named the “water-hammer theory” [7]. Chang and Nakagawa hypothesized that the loss of shock-absorbing capacity of the cisterna magna and subsequent increase in the pressure of the central canal wall resulted in syrinx formation in Chiari I malformation [8]. In rodent models of syringomyelia, rapid flow is observed from the spinal subarachnoid space into the perivascular spaces [9]. Syringomyelia results in increased fluid retention in the spinal cord and obstruction of the subarachnoid space, which might be a critical step in the development of the disease [10, 11]. Fluid outflow may be an important consideration in the pathogenesis of syringomyelia [12]; however, the mechanisms underlying the formation and enlargement of a syrinx or the source of the fluid are yet unclear.

The therapeutic decision is based on the resolution of the intrinsic mechanisms leading to syringomyelia [13]. Clinically, different approaches have been proposed to treat syringomyelia, including decompression of hindbrain disorders when syringomyelia is associated with Chiari malformation and resection of spinal tumors and intramedullary tumor, respectively [3, 13, 14]. Rarely, syringomyelia can resolve spontaneously via yet unknown mechanisms [15]. Cell transplantation has recently gained much attention as a potential treatment modality for syringomyelia [16]. A recent study reported a patient who suffered from syringomyelia-induced pain and received a transplant of uncultured umbilical cord-derived mesenchymal stem cells (MSCs) combined with surgery to manage Chiari malformation. Even though the initial purpose of the treatment was relieving pain, after two years of the stem cells treatment, the patient’s cavity had almost completely disappeared, and syringomyelia was deemed to be cured [17].

To investigate the mechanisms underlying syringomyelia pathogenesis, an in-depth study of its pathology is warranted. One of the significant pathological changes observed in syringomyelia patients is the dilation of the central canal on magnetic resonance imaging (MRI). The ependymal cells lining the central canal have been referred to as endogenous stem cells in mammalians [18, 19], and they are known to proliferate after spinal cord injury (SCI) [20–22]. We hypothesized the ependymal cells lining the central canal might contribute to the dilation of the central canal under syringomyelia condition. In the current study, we described the involvement of ependymal cells in syringomyelia pathogenesis and explored the underlying pathways. Our results showed that syringomyelia led to the activation of ependymal cells and the formation of tunnel-like protrusions in the central canal, resulting in its dilation and syrinx formation. Inhibition of TGFβR1-Smad3 was found to alleviate syringomyelia-induced central canal dilation, indicating the key role of TGFβR1-Smad3 in syringomyelia pathogenesis.

## Results

### Syringomyelia induces syrinx formation in the central canal, along with ependymal cell proliferation

**Fig. 1:**
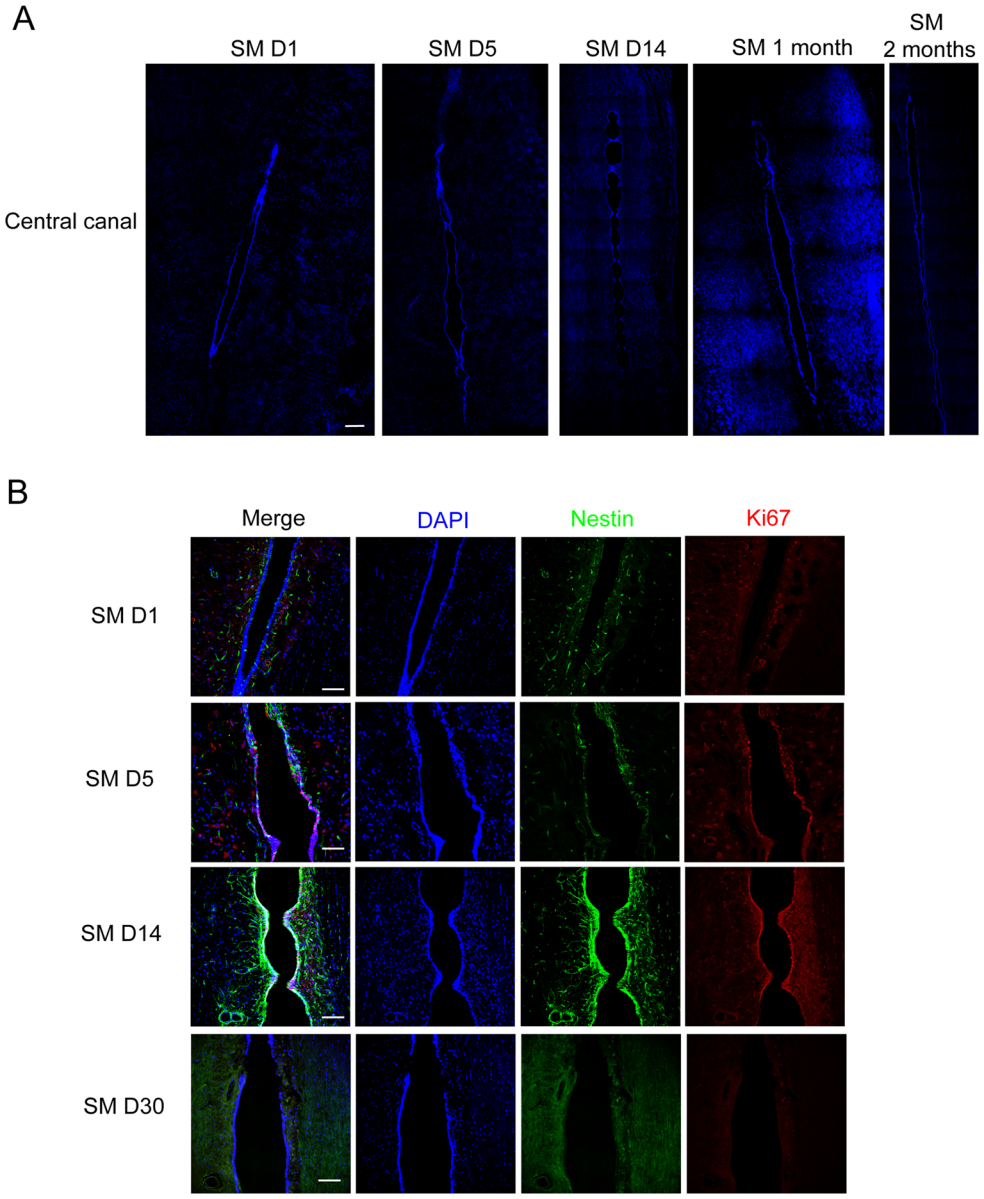
Dilation of central canal and activation of ependymal cells under syringomyelia. **A** Dilation of central canal at different time points of syringomyelia disease. Tunnel-like protrusions were obvious on day 14 of syringomyelia. SM, syringomyelia. DAPI, blue. Scale bar: 250 μm. **B** Ependymal cells lining central canal were activated. DAPI, blue; Nestin, green; Ki67, red. Scale bars: 100 μm

Clinically, most syringomyelia cases exhibit adverse impacts on the central canal. In our study, we established syringomyelia rat models according to a protocol proposed previously [23]. We observed syrinx formation on SM D5 (day 5 of syringomyelia) in the central canal by DAPI staining, with normal morphology on SM D1 and enlargement from SM D14 onward (Fig. 1A). We noticed that syrinx became obvious on SM D14, with many tunnel-like protrusions in the central canal. According to previous studies, ependymal cells of spinal cord remain quiet under normal conditions and are activated post-injury, such as spinal cord injury (SCI), inspiring us to detect whether the ependymal cells are activated by syringomyelia. Our results showed that ependymal cells located in the central canal co-expressed neural stem cell (NSC) marker Nestin and proliferative marker Ki67 (Fig. 1B). Nestin and Ki67 signals in the central canal appeared on days 1–5, peaked on SM D14, and diminished on SM D30 (Fig. 1B and Additional file 1: Fig. S1A), when syrinx kept stable. The results indicated that ependymal cells could be activated by syringomyelia.

### Tunnel-like protrusions in the central canal were ependymal cells

**Fig. 2:**
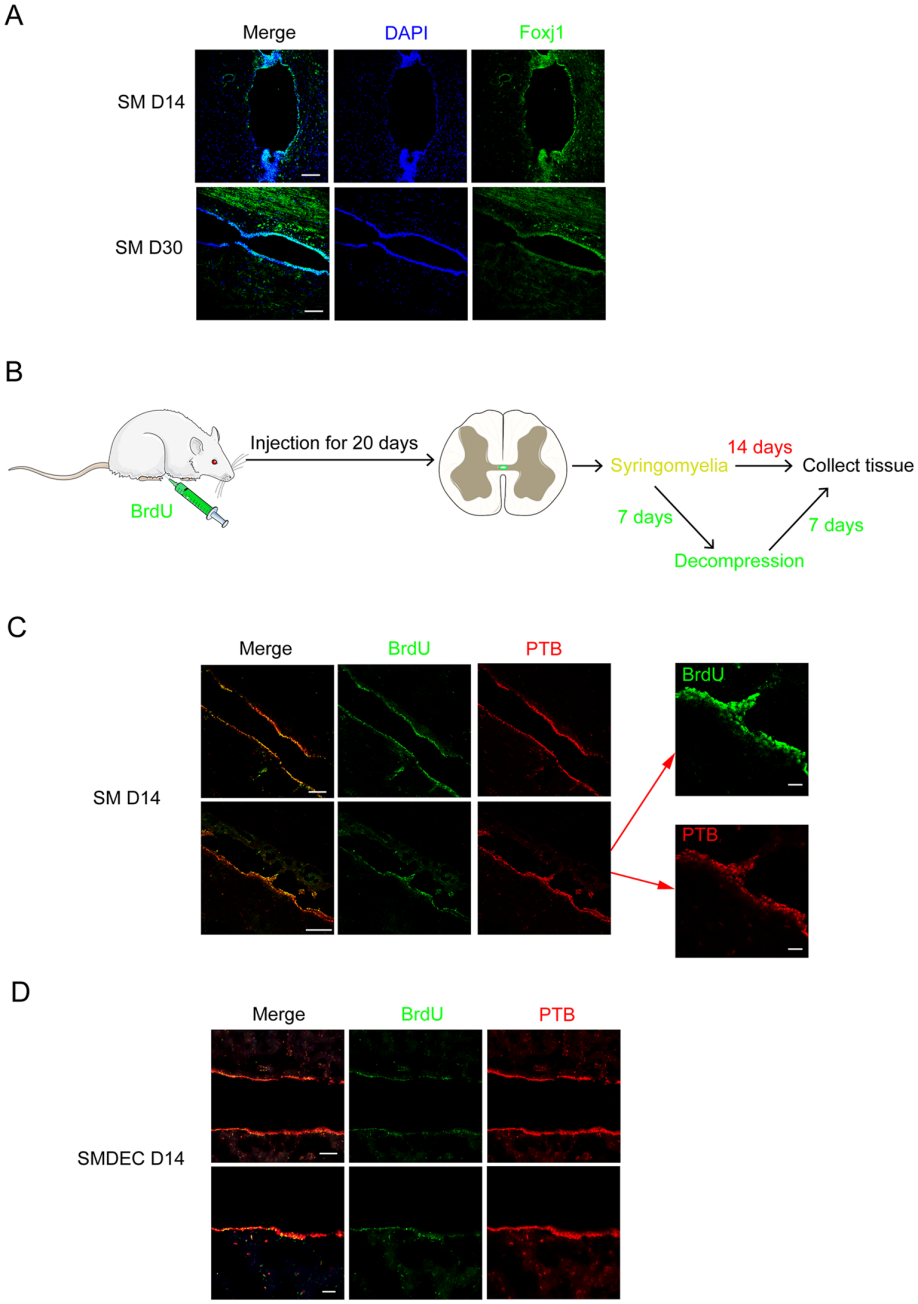
BrdU staining showed cells in protrusions of central canal under syringomyelia were ependymal cells. **A** Foxj1, an ependymal cell marker, was positive in the protusions on SM D14 and SM D30. DAPI, blue; Foxj1, green. Scale bars: 100 μm. **B** Experimental plan of BrdU injection. **C** BrdU^+^ cells were observed in protrusions on SM D14. The right panel were magnification of the left. DAPI, blue; BrdU, green; PTB, red. Scale bars in left: 100 μm. Scale bars in right: 20 μm. **D** BrdU^+^ cells were observed in protrusions after decompression. The spinal tissue in SMDEC D14 group were decompressed on SM D14 followed by collection and detection after another 7 days. DAPI, blue; BrdU, green; PTB, red. The lower panels were magnifications of the upper. Scale bar in upper: 100 μm. Scale bar in lower: 30 μm

Mechanisms underlying the formation of the tunnel-like protrusions was crucial in developing novel syringomyelia therapies. We speculated that the activation of ependymal cells under syringomyelia involved in the formation of protrusions. Our results indicated that SM D14 was the most appropriate time point when the proliferation/activation of ependymal cells peaked, so we focused on SM D14 observations. On SM D14 and SM D30, we found that the cells in the protrusions expressed Foxj1, a marker of ependymal cells (Fig. 2A). PTB was reported to be expressed in the ependymal cells [24] and we observed the co-expression of Foxj1 and PTB in the protrusions on SM D14 (Additional file 1: Fig. 1B and C). To further confirm the cell origins of the protrusions, we injected BrdU for 20 days consecutively to indicate inherent ependymal cells of spinal cord, followed by syringomyelia induction and decompression (Fig. 2B). We performed decompression after 7 days of syringomyelia, and collected spinal tissue in another 7 days (14 days in total) (Fig. 2B). Some PTB-expressing BrdU^+^ cells were observed in protrusions on SM D14 (Fig. 2C), as indicated by staining on SMDEC D14 (day 7 after decompression) (Fig. 2D). The results indicated that the tunnel-like protrusions were composed of ependymal cells.

### Ependymal cells might contribute to protrusions through migration along the central canal

**Fig. 3:**
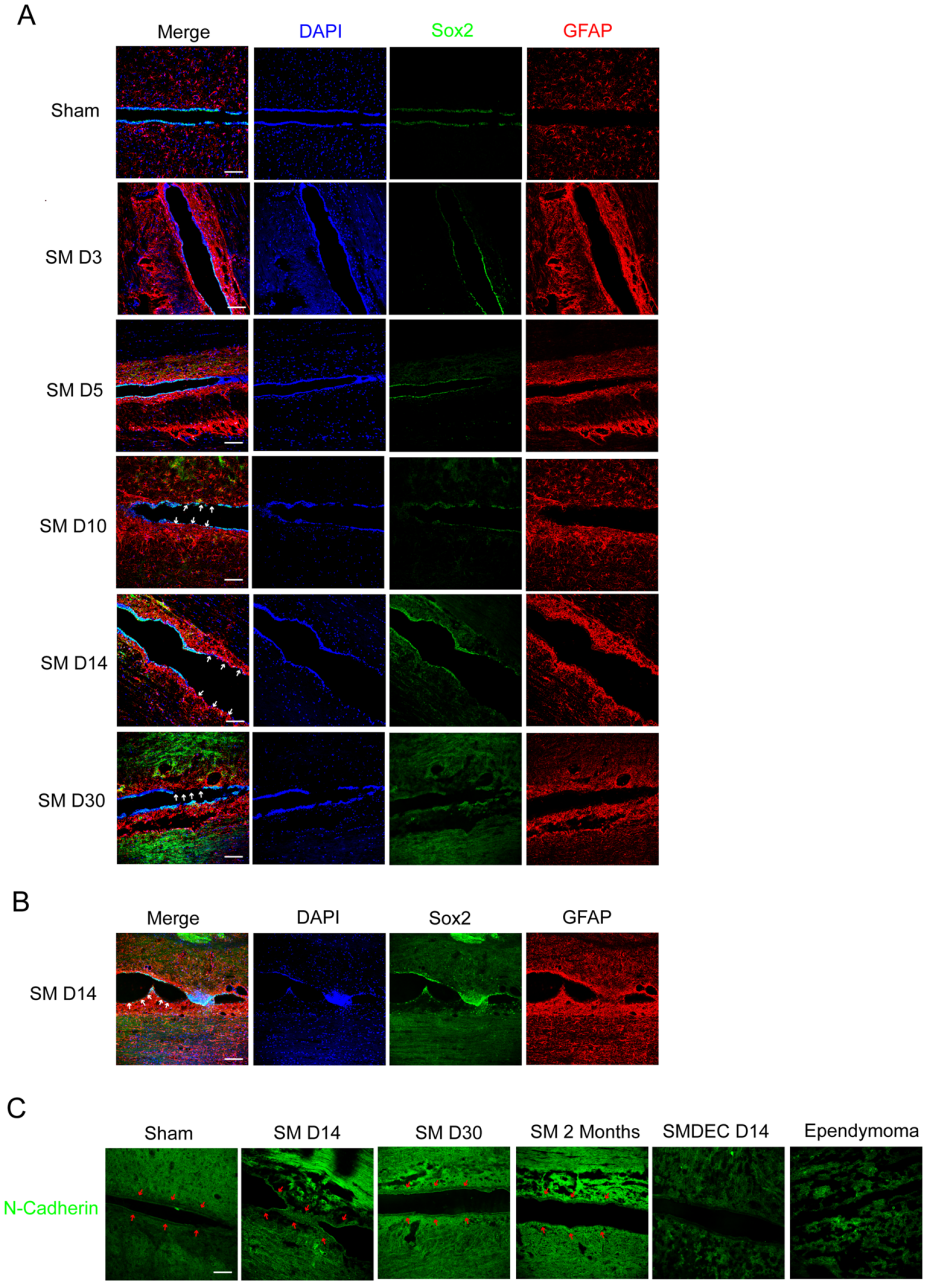
Some ependymal cells were dispeared along central canal. **A** Ependymal cells were particially dispeared form SM D10. The white arrows showed the diminished ependymal cells. DAPI, blue; Sox2, green; GFAP, red. Scale bars: 100 μm. **B** The magnification of ependymal cells on SM D14. The white arrows indicated cells lining central canal expressed GFAP but not Sox2. DAPI, blue; Sox2, green; GFAP, red. Scale bar: 100 μm. **C** Expression of N-cadherin under syringomyelia. The red arrows indicated the central canal. N-cadherin, green. Scale bar: 100 μm

According to previous reports, ependymal cells of the central canal were considered as a source of NSCs in spinal cord and were found to migrate after injury [25, 26]. Sox2, a marker of NSCs, was expressed in ependymal cells of intact/injured spinal cord and upregulated in injured spinal cord [27]. GFAP (a marker of astrocytes) was negative in ependymal cells and co-expressed with Sox2 in cells located close to the central canal or in the dorsal horns [28]. So we defined the ependymal cells in the central canal as Sox2^+^/GFAP^–^. In syringomyelia samples, we observed that some ependymal cells lining the central canal disappeared, such as on days 10, 14, and 30, with DAPI negative (Fig. 3A and B, as arrows indicated the disappeared cells). The Sox2-negative cells along central canal with expressed GFAP (Fig. 3B), indicating they were not ependymal cells. Three hypotheses might explain the partial deletion of ependymal cells: (1) The ependymal cells were deleted due to cell death; (2) The ependymal cells were reprogrammed into GFAP^+^ cells; (3) The ependymal cells left and migrated to other positions along central canal. BrdU staining revealed some BrdU^+^ cells in the protrusions (Fig. 2C), and we suspected the BrdU^+^ cells contributed the protrusions possibly through migration. In addition, we found that the central canal changed morphologies into several protrusions on SM D5 at different slice planes (Additional file 2: Fig. S2A), making migration of the ependymal cells feasible. Then, how did the ependymal cells migrate, and what about changes of the cell connections between ependymal cells, such as N-cadherin and E-cadherin? Both of N-cadherin and E-cadherin contributed in cell migration and the downregulation of E-cadherin was balanced by the enhanced expression of N-cadherin, resulting in a ‘cadherin switch’ that alters cell adhesion [29]. The N-cadherin interactions were weaker than E-cadherin interactions facilitated cell migration and invasion [30]. N-cadherin was not expressed by the ependymal cells in the sham group (Fig. 3C). However, in syringomyelia specimens, N-cadherin was expressed in ependymal cells, and diminished after decompression (Fig. 3C). Moreover, syringomyelia induced robust ependymal cell proliferation and protrusions which, like ependymal tumors, expressed N-cadherin (Fig. 3C) to some extent. The results suggested protrusions formation under syringomyelia might be similar with ependymal tumors which involved proliferation and migration of ependymal cells.

### TGFβR-Smad3 might play roles on protrusion formation and inhibit further enlargement of the syrinx

**Fig. 4:**
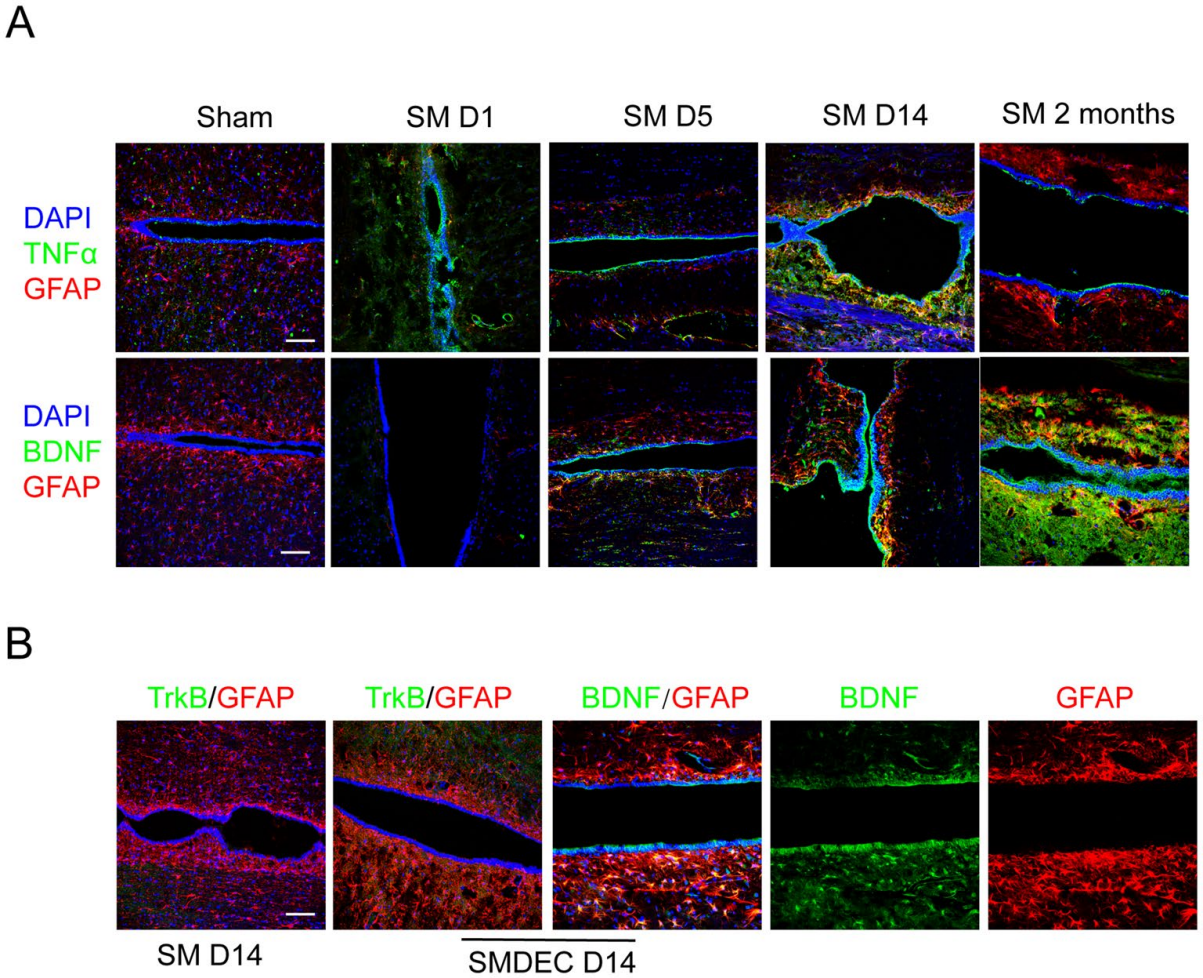
TNFα and BDNF expression under syringomyelia. **A** TNFα and BDNF were expressed in central canal. DAPI, blue; TNFα and BDNF, green; GFAP, red. Scale bars: 100 μm. **B** BDNF receptor TrkB was not expressed by central canal, whether decompression or not. DAPI, blue; TrkB and BDNF, green; GFAP, red. Scale bar: 100 μm

**Fig. 5:**
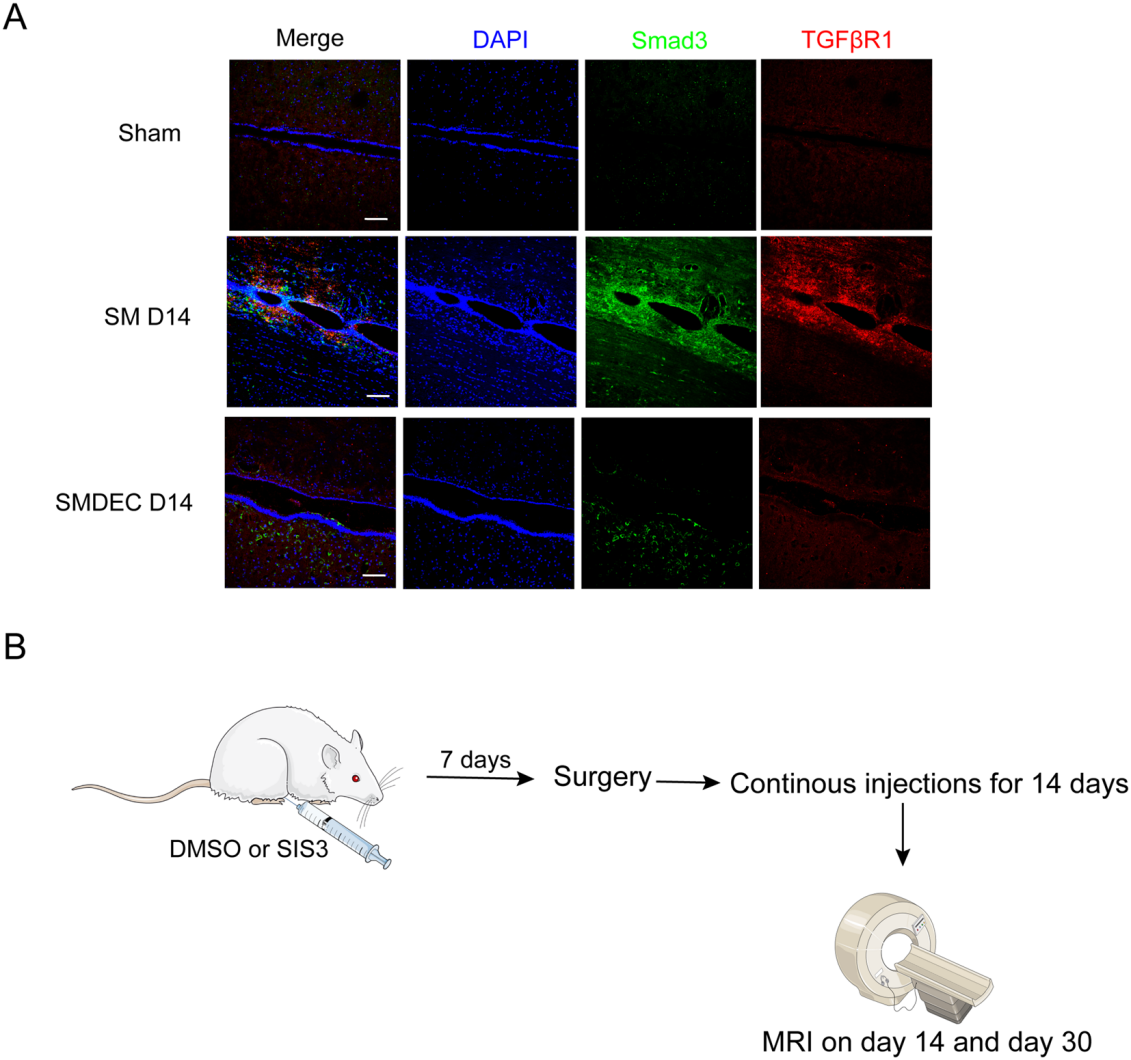
Expressions of TGFβR1 and Smad3 under syringomyelia. **A** TGFβR1 and Smad3 were expressed on SM D14, while not in sham or decompression groups. DAPI, blue; Smad3, green; TGFβR1, red. Scale bars: 100 μm. **B** Experimental plan of TGFβR1-Smad3 inhibition in vivo

**Fig. 6:**
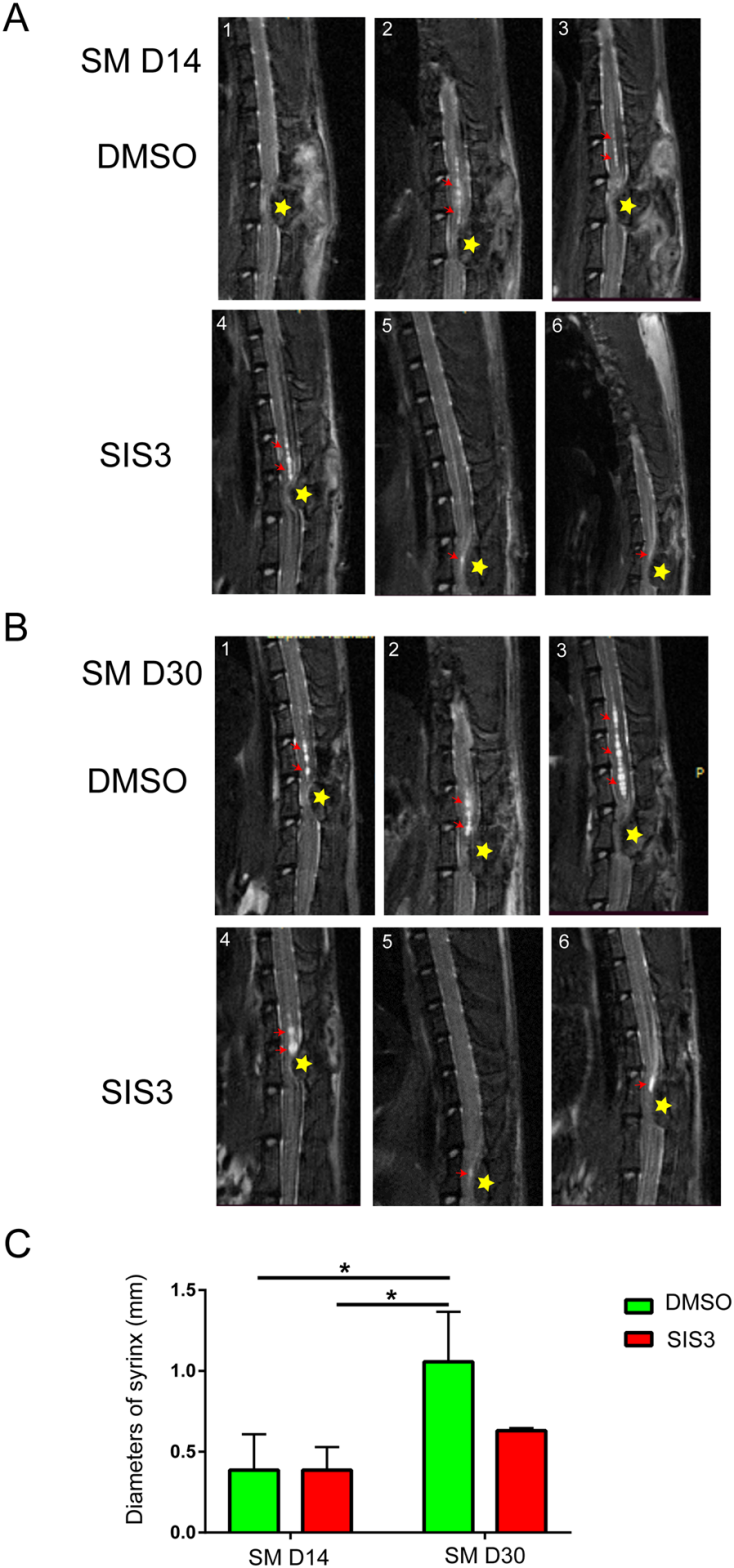
MRI of DMSO and SIS3 treated rats. **A** MRI showed no significant difference of syrinx size between the two groups on SM D14. **B** MRI showed that the size of syrinx was decreased after SISI3 treatment on SM D30. Each number in **A** and **B** indicated the individual rat, the red arrows indicated the syrinx in central canal, and the yellow stars showed the compressed positions by cotton strips. **C** Statistics of syrinx size of the two groups. *p < 0.05 and **p < 0.01

**Fig. 7:**
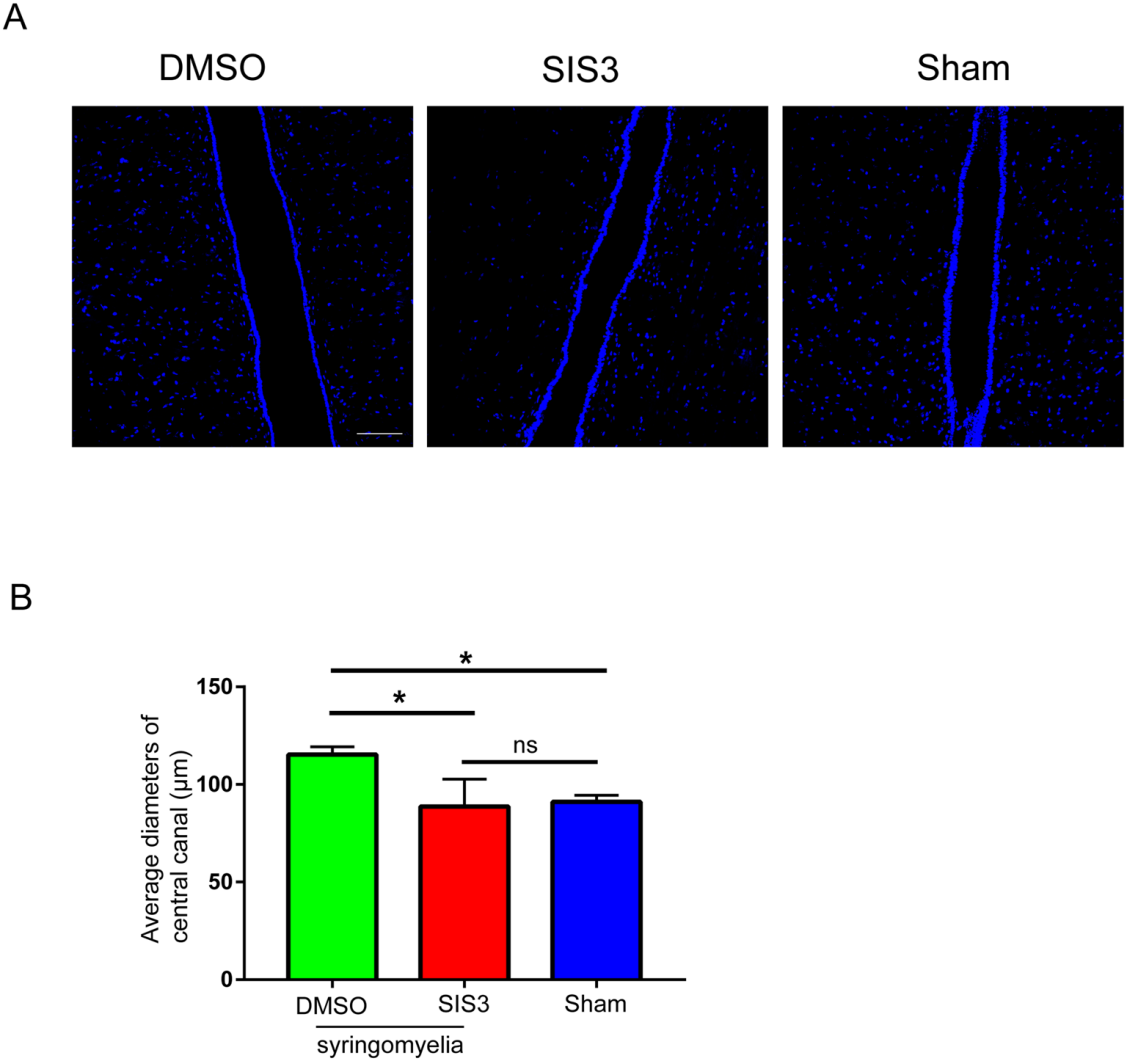
DAPI staning showed the reduced diameters of central canal in SIS3 group. **A** Inhibition of dilation of central canal in SIS3 group. DAPI, blue. Scale bar: 100 μm. **B** Statistics of average diameters of central canal in the two groups. *p < 0.05 and **p < 0.01

The microenvironment play important roles in neural degeneration disease such as SCI [31–33] and we suspect it may be involved in protrusion formation induced by syringomyelia. In the central canal, we detected the expression of IL4 (interleukin 4), IL6, NT3 (neurotrophin-3) and did not found positive signals except TNFα (tumor necrosis factor-α) and BDNF ( brain derived neurotrophic factor). TNFα was expressed constitutively and stably from SM D1, while BDNF which was involved in sensory function, was expressed from SM D5 and peaked on SM D14 (Fig. 4A) that was consistent with the activation of ependymal cells. However, the expression of BDNF did not change after decompression on SM D14 (Fig. 4B). Moreover, irrespective of decompression, the ependymal cells did not express the BDNF receptor TrkB (Tyrosine kinase receptor B) (Fig. 4B), indicating that BDNF might not be involved in protrusion formation. TGFβR (transforming growth factor beta β receptor)-Smad3 signal has been reported as an important pathway controlling cell migration and EMT (epithelial–mesenchymal transition) in various tumors [34–41], so we investigate this pathway’s role on the migration of ependymal cells in the current study. Our results showed negative TGFβR-Smad3 activity in the sham group, high activity in SM D14 specimens, and diminished activity post-decompression on SMDEC D14 (Fig. 5A). To confirm the role of the TGFβR-Smad3 axis on protrusion and syrinx formation, we injected DMSO and SIS3, a Smad3 inhibitor, into rats with syringomyelia by intraperitoneal injection respectively in vivo (Fig. 5B). DAPI staining showed syrinx was reduced in SIS3 group (Additional file 2: Fig. S2B) on SM D5. In addition, SIS3 treatment downregulated N-cadherin and upregulated E-cadherin expression (Additional file 2: Fig. S2C), indicating the inhibitory effects of SIS3 on TGFβR-Smad3 pathway, which enhanced N-cadherin and reduced E-cadherin expression involved in EMT process. MRI showed that both Control (DMSO injection) and SIS3 groups presented syrinx in the central canal on SM D14 (Fig. 6A) without significant difference. On SM D30, we found that the syrinx was enlarged in the Control group compared with the SIS3 group (Fig. 6B and C), in consistant with DAPI staining (Fig. 7), indicating that SIS3 inhibited further enlargement of the syrinx.

## Discussion

Surgical treatment is the only treatment method for syringomyelia that focuses on the cause of the cavity. It helps reduce the pressure of the subarachnoid space via a shunt that is mainly used to draw fluid out from the cavity [14, 42, 43]. Cell therapy can be considered an alternative therapy to treat syringomyelia. Animal experiments have shown good therapeutic effects of MSCs and NESCs (neuroepithelial-like stem cells) injection on syringomyelia [16, 44, 45]. Previous case reports have indicated that cell transplantation facilitates a reduction in syrinx and alleviation of disease [46, 47]. However, pains and somatic sensory disturbances due to syringomyelia remain an unsolved issue for many patients [48].

### Establishment of a perfect syringomyelia animal model is required

The decision regarding appropriate clinical therapy depends on the mechanisms underlying the disease. Animal models are the best tools for foundational research of desease. In the current study, we employed female SD rats because they are easier to care for post-op and exhibit less aggression than male rats [49–51]. Sex plays a significant role in the prevalence of Chiari I malformation and syringomyelia (female:male = 1.3:1 ~ 4:1) [52]. Previous reports have shown a female predominance in both pediatric and adult Chiari I malformation populations [53, 54]; syringomyelia is found more commonly in girls than in boys [55]. Even so, males should be validated among syringomyelia models in future experiments.

Since syringomyelia is rare, scientists are trying to establish animal models for syringomyelia through epidural compression, quisqualic acid, and kaolin injections into the spinal cord to explore the pathogenesis and adequate therapies for the disease [56, 57]. MRI, as a gold standard for definite SCI, has been the most commonly used tool for diagnosing syringomyelia [58–60]. However, a good animal model should stimulate not only pathological characteristics but also the symptoms of the disease. Separation of pain and fever caused by syringomyelia is rarely imitated in animals, partly due to the physiological differences between humans and non-human organisms, such as rats and mice. In clinical, doctors could understand the recovery of patients through interrogation, while scientists could not in animal study. Therefore, improved animal models and developed medical testing instruments are urgently required for a more effective investigation of syringomyelia.

### The relevance of the syrinx reduction to recovery following syringomyelia

Clinically, syrinx were decreased from MRI after treatment with syringomyelia, and case reports showed the symptoms of syringomyelia responded to reduction of the syrinx cavities [61–64]. The size of the syrinx was generally reduced after decompression with an improvement of the symptoms, however, the symptoms was improved clinically with no change of syringe size after decompression treatment [65]. In addition, few studies describe the management of residual syrinx after decompression, and there is general agreement that the aim of clinical treatment is to restore relatively unimpeded flow of cerebrospinal across the craniocervical junction [66]. Large holocord syrinx may induce permanent symptoms of SCI even with adequate decompression and reduction of the syrinx [66]. In our study, inhibition of TGFβR-Smad3 signaling alleviated the enlargement of syrinx, however, it is possible to result in off-target effects or incomplete inhibition of the signal. Activation of TGFβR-Smad3 pathway and knockdown/knockout of TGFβR or Smad3 by using more syringomyelia animal models would be helpful for detection of the syrinx enlargement under syringomyelia in the future experiments. In addition, further in-depth research about the mechnisms of syrinx generation is needed trying to selected optimal treatment on syringomyelia.

## Conclusions

In this study, we found the ependymal cells of spinal cord proliferated and formed tunnel-like protrusions in central canal possibly through migration. Inhibiting TGFβR-Smad3 pathway might play roles on slowing down the dilation of central canal and the progress of syringomyelia disease, providing therapy potential on syringomyelia in clinical.

## Methods

### Animals and ethical approval

Female 8-week-old Sprague–Dawley (SD) rats (Charles River, Beijing, China, n = 78) were used in this study. All animals were housed in temperature- and humidity-controlled animal quarters with a 12-h light/dark cycle. All of the animals were divided randomly for different assay: pathological assay (n = 54), BrdU assay (n = 6) and inhibitors administration (n = 18). All animal experiments were performed in accordance with the Chinese Ministry of Public Health Guide and the US National Institutes of Health Guide for the care and use of laboratory animals. All experimental procedures were approved and performed in accordance with the standards of the Experimental Animal Center of Xuanwu Hospital Capital Medical University (XW-20210423-2).

### Animal surgery

Anesthesia induction was performed in an anesthesia chamber using 2% enflurane (Yipin Corp., Hebei, China) in 70% nitrous oxide and 30% oxygen (Bickford veterinary anesthesia equipment model no. 61010; AM Bickford, Inc., Wales Center, NY, USA) through a nose cone. All surgical procedures including syringomyelia induction and decompression of rats with syringomyelia were performed in a sterile field according to a protocol proposed previously [23]. Briefly, an approximately 3 cm skin incision was made, and T12, T13, and L1 vertebral laminae were completely exposed. Aseptic cotton strips weighing 1.5 mg were gently stuffed into the extradural space below the T13 lamina from the incision of the ligamentum favum in the T12–13 lamina space under a surgical microscope (OPMI Pico, Carl Zeiss, Oberko Chen, Germany). For decompression, the cotton strip was carefully cut of using micro-tweezers and micro-scissors. All rats were kept and observed in conventional and clean rat houses. After the surgery, cefuroxime sodium (Sinopharm group Zhijun pharmaceutical co. LTD, China) was injected intraperitoneally every 8 h for 1 week.

### 5-BrdU administration

5-BrdU (MCE, BrdU in short) disloved in prue water adding 50% PEG300 (polyethylene glycol, Selleck) was daily adiministrated by intraperitoneal injection (50 mg/Kg) for 20 days before syringomyelia models induction or decompression of syringomyelia rats and terminiated in the following days during the experiments (n = 6).

### SIS3 administration

Each rat (n = 9 in each group) received SIS3-Hcl (Selleck, SIS3 in short) disolved in 10% DMSO by intraperitoneal injection (2.8 mg/Kg) 7 days before syringomyelia induction. 10% DMSO was injected as control. DMSO and SIS3 injections were continued until the end of the experiment. All of the rats in the two groups were not decompressed after syringomyelia induction.

### MRI in vivo

MRI was performed using a 7.0 Teslan MRI scanner (PharmaScan 7 T, Bruker Corp., Karlsruhe, Germany) with 400 mT/m gradients in the Animal Imaging Experimental Center at Capital Medical University. The rats were placed on the table in the prone position with two restraining belts to fix the trunk. General anesthesia was induced via 4% isoflurane in oxygen before scanning and maintained by 2% isoflurane in oxygen via a rat mask during scanning. The body temperature, heart rate, and respiration of the rats were closely monitored during imaging. Once rapid whole-body localization scans were performed in all three planes, sagittal and axial T2-weighted images were acquired with the operation area as the center using a fat-saturated RARE sequence. A rat volume coil with a diameter of 89 mm was used for transmission and to obtain data. Imaging parameters for sagittal acquisition were as follows: TR/TE = 3000/33 ms, matrix size = 256 × 256, field of view (FOV) = 60 × 40 mm^2^, slice thickness = 600 µm with no gap, number of slices = 10, NEX = 8, and resolution = 0.147 × 0.147 × 1 mm^3^. The imaging parameters for axial acquisition were as follows: TR/TE = 4500/33 ms, matrix size = 256 × 256, FOV = 60 × 40 mm^2^, slice thickness = 1 mm with no gap, number of slices = 30, NEX = 8, and resolution = 0.147 × 0.147 × 1 mm^3^. Each MRI scan took approximately 12 min. The anteroposterior (AP) diameter of the syrinx every 1 mm from the full length of the syrinx was measured in sagittal T2-MRI images. The largest diameter of these was selected, and the AP diameters of the spinal cord in the same plane were measured to calculate the diameter ratio. All measurements were made using the Horos software platform (v3.3.5, https://horosproject.org).

### Tissue collections

Rats were perfused with 4% paraformaldehyde (PFA) following euthanization by pentobarbital sodium (150 mg/kg IP). The spinal cord from T6 to T10 was harvested carefully to retain its integrity, fixed in 4% PFA for 48 h at 4°C, and transferred to 20% and 30% sucrose for 24 h. The segments were then embedded in an optimal cutting temperature compound (OCT), cut into 20-µm thick sections (Leica Microsystems), and stored at − 80°C.

### Immunofluorescence

The spinal cord sections were first pretreated in 0.3% Triton X-100 in phosphate-buffered saline (PBS, pH 7.4) for 20 min, followed by incubation in 10% bovine serum albumin (BSA) for 1 h at room temperature (RT). The sections were then incubated with primary antibodies overnight at 4 °C. The primary antibodies used were anti-BDNF (Bioss, bs-4989R), anti-E-cadherin (Proteintech, 20874-1-AP), anti-Foxj1 (abcam, ab178847), anti-GFAP (Abcam, ab4674), anti-Ki67 (Millipore, AB9260), anti-N-cadherin (Proteintech, 66219-1-lg), anti-Nestin (Millipore, MAB353), anti-PTB (Proteintech, 12582-1-AP), anti-Smad3 (abcam, ab40854), anti-Sox2 (Santa Cruz, sc-365823), anti-TGFβR1 (abcam, ab31013), and anti-TNFα (Immunoway, YT4689). The slides were washed three times with PBS and subsequently incubated with conjugated secondary antibodies (Jackson ImmunoResearch Laboratories) for 2 h at RT. DAPI (1 mg/mL) was used to counterstain nuclei. The images were captured using a confocal microscope Leica SCN400 Slide Scanner (Leica Microsystems) using the same settings, such as voltage, background reduction, and other parameters.

### Statistical analysis

All the data were expressed as mean ± standard deviation (mean ± SD). Statistical evaluations were conducted with GraphPad Prism 5 (GraphPad Software, San Diego, USA). Differences between groups were compared using the two-tailed Student’s t-test or ANOVA. Differences were considered significant when *p < 0.05 and **p < 0.01.

## Supplementary Information

**Additional file 1: Figure S1.** Tunnel-like protrusions formed in central canal on SM D14. Ependymal cells were activated on SM D14. DAPI, blue; Nestin, green; Ki67, red. Scale bar: 500 μm. Foxj1 staining indicated ependymal cells in protrusions. DAPI, blue; Foxj1, green; PTB, red. Scale bar: 500 μm. The white dot lines showed the protrusions. Magnification of domains defined by white dot lines in . DAPI, blue; Foxj1, green; PTB, red. Scale bar: 100 μm.

**Additional file 2: Figure S2.** Morphologies and stainings of central canal on SM D5 with or without SIS3 administration. Central canal on different planes of section on SM D5. Central canal morphologies after SIS3 treatment on SM D5. DAPI, blue. N-cadherin and E-cadherin stainings in DMSO and SIS3 groups. DAPI, blue; N-cadherin, green; E-cadherin, red. Scale bars: 100 μm.

## Data Availability

The data of the current study are presented in the figures. If necessary, the data that support the findings of this study are available from the corresponding author upon reasonable request. Source data are provided with this paper.

## Abbreviations

ANOVA: Analysis of variance
BDNF: Brain derived neurotrophic factor
BSA: Bovine serum albumin
CSF: Cerebrospinal fluid
IL: Interleukin
EMT: Epithelial–mesenchymal transition
MRI: Magnetic resonance imaging
MSCs: Mesenchymal stem cells
NESCs: Neuroepithelial-like stem cells
NSC: Neural stem cell
NT3: Neurotrophin-3
OCT: Optimal cutting temperature compound
PBS: Phosphate-buffered saline
PEG: Polyethylene glycol
PFA: Paraformaldehyde
RT: Room temperature
SCI: Spinal cord injury
SD: Sprague–Dawley
SM: Syringomyelia
SMDEC: Decompression after Syringomyelia
TGFβR: Transforming growth factor beta β receptor
TNFα: Tumor necrosis factor-α
TrkB: Tyrosine kinase receptor B
