# Supplementary figures and images for: Suppression of TGFβR-Smad3 pathway alleviates the syrinx induced by syringomyelia
Source: Cell Biosci. 2023 May 29;13:98. doi: 10.1186/s13578-023-01048-w (PMC10226251; doi:10.1186/s13578-023-01048-w)

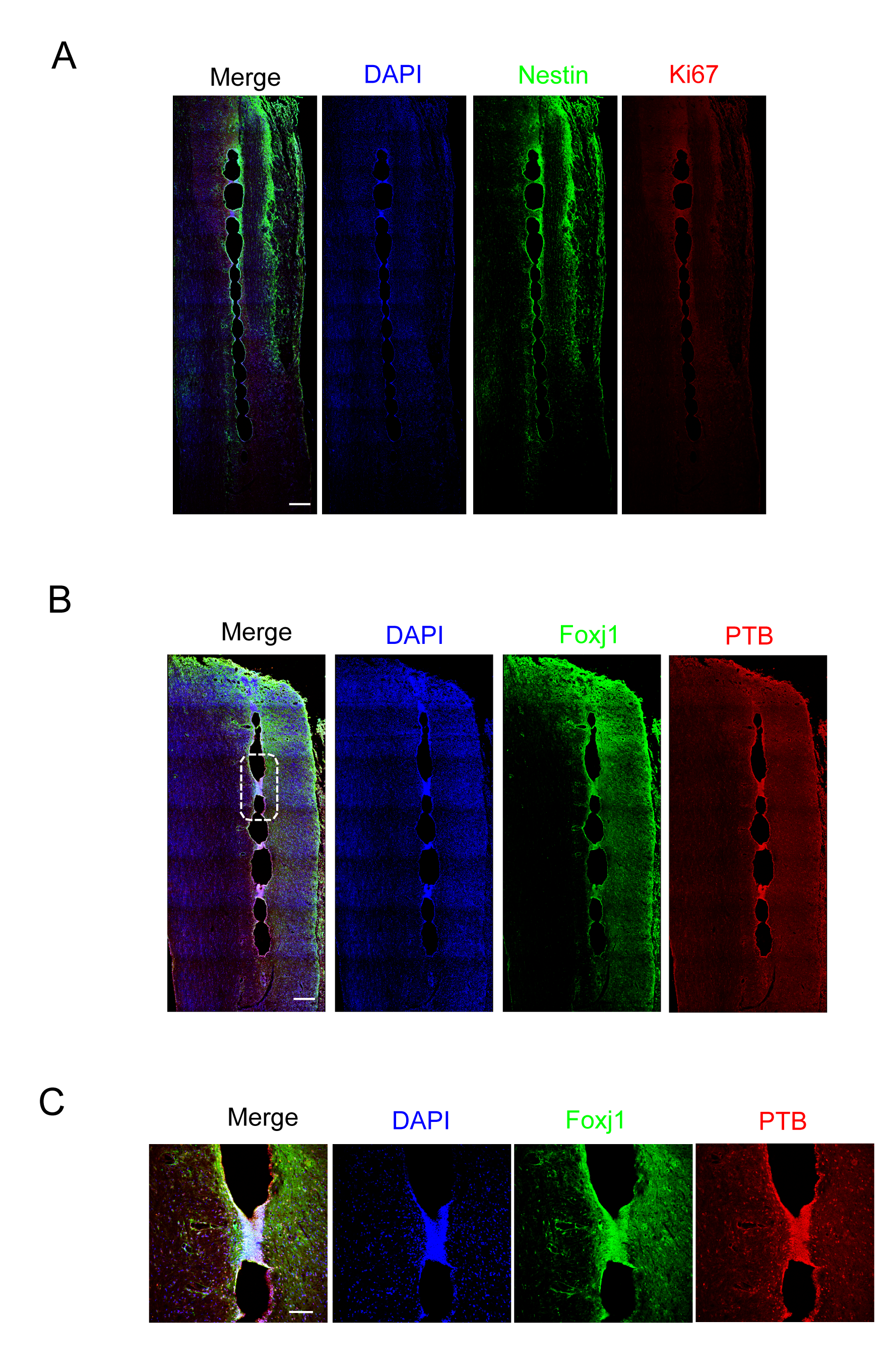

Supplement: Supplementary file 1 — Additional file 1: Figure S1. Tunnel-like protrusions formed in central canal on SM D14. Ependymal cells were activated on SM D14. DAPI, blue; Nestin, green; Ki67, red. Scale bar: 500 μm. Foxj1 staining indicated ependymal cells in protrusions. DAPI, blue; Foxj1, green; PTB, red. Scale bar: 500 μm. The white dot lines showed the protrusions. Magnification of domains defined by white dot lines in . DAPI, blue; Foxj1, green; PTB, red. Scale bar: 100 μm. [file 13578_2023_1048_MOESM1_ESM.tif]

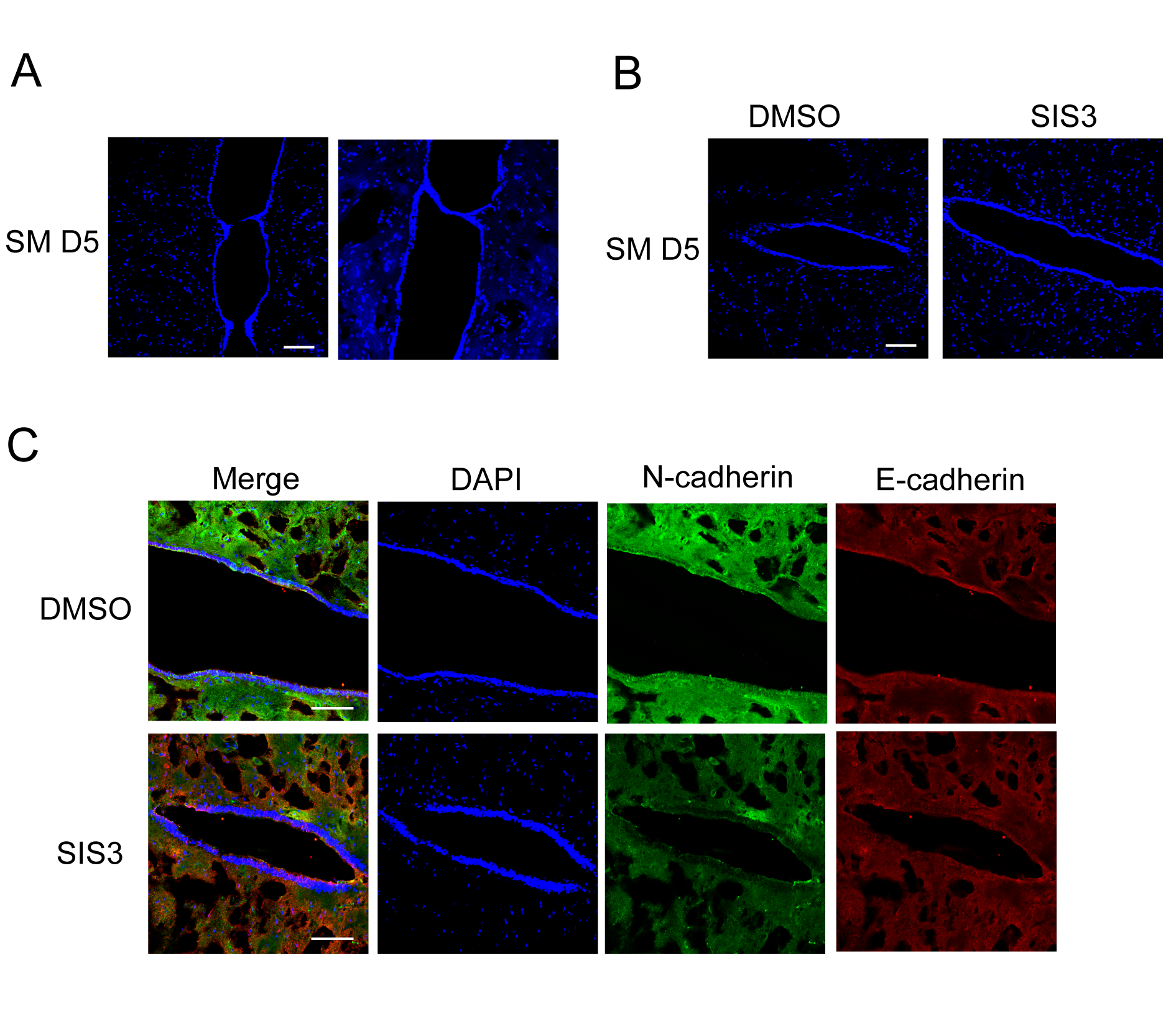

Supplement: Supplementary file 2 — Additional file 2: Figure S2. Morphologies and stainings of central canal on SM D5 with or without SIS3 administration. Central canal on different planes of section on SM D5. Central canal morphologies after SIS3 treatment on SM D5. DAPI, blue. N-cadherin and E-cadherin stainings in DMSO and SIS3 groups. DAPI, blue; N-cadherin, green; E-cadherin, red. Scale bars: 100 μm. [file 13578_2023_1048_MOESM2_ESM.tif]
